# Information Management at a Health Services Research Organization in Toronto, Ontario, Canada: Moving from Identifiable Data to Coded Data

**DOI:** 10.23889/ijpds.v1i1.307

**Published:** 2017-04-19

**Authors:** Lisa Thurairasu, Nelson Chong

**Affiliations:** Institute for Clinical Evaluative Sciences (ICES)

## Objectives

A health services research organization in Toronto, Ontario, Canada conducts population-based research to improve the health of Canadians in seven main areas: (1) cancer, (2) cardiovascular disease, (3) chronic disease and pharmacology, (4) health system planning and evaluation, (5) kidney, dialysis and transplantation, (6) mental health and addictions, and (7) primary care and population health. The Information Management (IM) team within the Data Quality and Information Management (DQIM) department at our non-profit organization is an integral component for upholding privacy and confidentiality policies and procedures while facilitating quality research using different types of data such as health administrative, third-party, primary data collection, and electronic medical records (EMR).

## Method

The IM team is responsible for receiving data, encoding direct personal identifiers, screening for unnecessary identifiers, performing probabilistic data linkage when necessary, importing the data to the Research Analytics Environment (a client/server Linux-based system), and destroying the data according to the terms stipulated in the executed data sharing agreement. The purpose of the presentation is to detail the above steps of processing data to protect individuals' identities yet preserve the usefulness of carrying out research. The presentation will include aspects from importing data into SAS to storage and encoding of personal identifiers to probabilistic data linkage, which involves maximizing linkage with other datasets at the organization. Linking data at the organization involves the encryption or encoding of health card numbers to "Key Numbers".

## Results

The processing practices used at the organization comply with Canadian privacy laws such as the Personal Health Information Protection Act (PHIPA) as well as organizational policies and Research Ethics Board approvals. The approaches used to conceal individual identities yet allow linkage to various data sources can be modelled by other health agencies, ministries, and non-health related organizations that work with sensitive data but face challenges in maintaining both privacy and research quality. Our organization strives to make processing as efficient as possible and create maximum linkability to the various data sources in house while upholding privacy and confidentiality.

